# Ultrasonic vocalization of pup and adult fat-tailed gerbils (*Pachyuromys duprasi*)

**DOI:** 10.1371/journal.pone.0219749

**Published:** 2019-07-29

**Authors:** Alexandra S. Zaytseva, Ilya A. Volodin, Olga G. Ilchenko, Elena V. Volodina

**Affiliations:** 1 Department of Vertebrate Zoology, Faculty of Biology, Lomonosov Moscow State University, Moscow, Russia; 2 Scientific Research Department, Moscow Zoo, Moscow, Russia; Tierarztliche Hochschule Hannover, GERMANY

## Abstract

Ultrasonic vocalizations (USVs) of laboratory rodents indicate animal emotional arousal and may serve as models of human disorders. We analysed spectrographically USV calls of pup and adult fat-tailed gerbils *Pachyuromys duprasi* during 420-s tests, including isolation, touch and handling. Based on combination of six different USV syllable contour shapes and six different note compositions, we classified 782 USV syllables of 24 pups aged 5–10 days to 18 types and 232 syllables of 7 adults to 24 types. Pups and adults shared 16 of these 26 USV types. Percentages of USV syllables with certain contour shapes differed between pups and adults. The contour shape and note composition significantly affected most acoustic variables of USV syllables in either pups or adults. The 1-note USV syllables were most common in either pups or adults. Pup USV syllables were overall longer and higher-frequency than adult ones, reminiscent of the USV ontogenetic pathway of bats and distinctive to rats and mice. We discuss that the USV syllable types of fat-tailed gerbils were generally similar in contour shapes and note compositions with USV syllable types of mice and rats, what means that software developed for automated classifying of mice ultrasound might be easily adapted or re-tuned to gerbil USV calls. However, using fat-tailed gerbils as model for biomedical research including control of USV vocalization is only possible since 6^th^ day of pup life, because of the delayed emergence of USV calls in ontogeny of this species.

## Introduction

Ultrasonic vocalizations (USV) indicate emotional arousal and impairments in rodents [1–5]. Classifying discomfort-related ultrasonic calls is an important prerequisite for their applicability as indicators of emotional arousal and welfare [2,6–13]. In addition, rodent USV are widely used in biomedical experiments modeling human affective and communicative disorders [11,14–18].

Although current biomedical research is primarily based on studying laboratory mice and rats [1,19], other rodent species may possess by peculiar features, which would make them especially suitable as test animals for modeling certain diseases. For example, the Mongolian gerbil *Meriones unguiculatus* represents an especially convenient animal model for human epilepsy [20,21] because of absence of connecting arteries between the basilar and carotid systems [22]. The Norvay rat *Rattus norvegicus* model is the exclusive model of vocal negative and positive emotional correlates embedded in the 22 kHz and 50-kHz USV calls [1,23], which are not clearly distinctive in the mice model [4]. The prairie vole *Microtus ocrogaster* is a particularly useful animal model for examining social effects on the relationship between heart rate and USV fundamental frequency because of a low and variable heart rate and the very simple acoustic structure of the USV calls [24,25] as well as an unusually large functional and anatomical representation of auditory cortex [26]. Further analyses of USV in different rodent species (hamsters, voles, gerbils) are therefore advantageous for searching new exclusive animal models for various behavioural and medical research [27].

Rodents produce USV in various contexts [28,29]. Pup rodents produce USV during social play [30–32], isolation from the lactating mother and littermates [12,33–40], pain [41] and handling [34,39,42].

Adult rodents produce USV at pair bonding [43–51], novelty [52,53], territorial defense [54], social encounters [55–57], alarm [52,58–61], spontaneously during various everyday activities [62], echo-based orientation [63,64] and at pain [65]. During isolation procedure in the lab, adult rodents either emit USV, as do e.g. *Glaucomys* flying squirrels [66], domestic mice *Mus musculus* [4] and *Scotinomys* singing mice [67], or may remain silent, as e.g. Mongolian gerbils [68,69]. During handling in the lab, the USV calls were reported in adult laboratory rats [70].

Distinctive to the audible calls, which are generated by passive flow-induced vocal fold oscillations [71], studies on mice and rats show that the most likely mechanism for producing rodent USV is so called “jet” or “whistle”, mechanism [72–76]. The jet mechanism generates USV calls because of an obstruction in the path of air jet, such as sharp edge, a hole, or a side branch [72]. The jet mechanism is probably responsible for the variability of the fundamental frequency (f0) contours (flat, chevron, wave, upward, downward) observed in rodent USV syllables [6–8,35,73,77–79]. In addition, the USV syllable contours can be either continuous (1-note) or can be broken to 2, 3, or more notes by frequency jumps [6,7,10].

Gerbils are widely used as animal models in biomedical research [80–83]. The fat-tailed gerbil (*Pachyuromys duprasi*) represents a perspective animal model for studying USV calls [39]. This is a North African rodent species kept at laboratories and zoos [39,80,84]. Fat-tailed gerbils were used for studying ear morphology and hearing [85–87], tropical diseases [80,81], thermoregulation [88,89] and physical development [84]. The fat-tailed gerbil is a medium-sized gerbil, with body mass in adults (with breeding experience) of 60.0 ± 24.3 g and head length of 39.6 ± 2.1 mm, without significant differences between sexes [84]. In 7-d pups, body mass is 5.3 ± 0.7 g and head length is 18.4 ± 0.8 mm [84].

Pup and adult fat-tailed gerbils produce both audible and USV calls [39,55]. In adults, the low-frequency wideband chirrs along to harmonic audible calls and USV calls up to 60 kHz were registered when two unfamiliar animals (male-male, female-female or male-female) were placed together in the one cage [55]. In pups, both audible and USV calls occur during isolation and handling procedure [39]. Variables of pup “joint calls” (representing a sum of all USV calls emitted within a test trial with cut-off inter-call intervals) reflect a degree of discomfort in 8-40-d fat-tailed gerbil pups [39].

Whereas the audible calls of fat-tailed gerbils emerge since 1^st^ day of pup life, first USV calls emerge only since 5^th^ day of pup life, with maximum of ultrasound emission in 12-15-d pups [39]. This ontogenetic delay of USV emergence is unusual. In other rodents (gerbils, mice, California mice, voles, rats, hamsters), the isolation-induced USV calls emerge since 1^st^-3^rd^ day of life, depending on the species [33,37,38,42,90–102].

The classifying of USV syllables to types and measuring their acoustic variables have not yet been done for fat-tailed gerbils. The aim of this study was to develop a categorization of fat-tailed gerbil USV syllables and to compare their acoustics between pups and adults. As soon as USV calls of fat-tailed gerbils are missing until 5^th^ day of pup life [39], we selected for the comparative analyses of pup and adult USV calls only those acoustic recordings collected from 5-9-d pups.

## Material and methods

### Ethics statement

This study was part of the research program of the Scientific Research Department of Moscow Zoo. All the four authors are zoo staff members, so no special permission was required for them to work with animals in Moscow Zoo. All study animals belonged to the laboratory collection of Moscow Zoo. The experimental procedure has been approved by the Committee of Bio-ethics of Lomonosov Moscow State University, research protocol # 2011–36. We adhered to the ‘Guidelines for the treatment of animals in behavioural research and teaching’ (Anim. Behav., 2006, 71, 245–253) and to the laws on animal welfare for scientific research of the Russian Federation, where the study was conducted. No one single animal suffered due to data collection.

### Study site and subjects

The USV calls were collected from members of a captive colony of fat-tailed gerbils at Moscow Zoo, Moscow, Russia, in May-July 2013 and in June-August 2014. Our study animals were 40 6-10-d pups (17 males and 23 females from 11 litters) and 20 adults with breeding experience (10 males, 10 females). Study pups were sexed between 12 and 19 days of age, on average at 15.1 *±* 2.0 days of age based on the appearance of nipples in females [84,103]. The small size of pups also prevented individual chip marking for ethical reasons until 18–20 days of age.

Before parturition, females were checked once a day for the appearance of a litter, and birth dates as well as the number of pups were recorded. The 11 study litters, containing in total 40 study pups, originated from 10 different mothers: nine mothers with one litter per female and one female gave birth to two litters. The litter size varied from 2 to 6 pups (mean ± SD = 4.00 ± 1.34). The day of birth was considered zero day of pup life.

### Animal housing

The animals were kept under a natural light regime at room temperature (24–26°C), in family groups consisting of two parents and littermates, because a male is non-aggressive to pups and the appearance of a second litter is possible without separation of the first one [39,84]. The animals were housed in wire-and-glass cages of 51x42.5x41.5 cm, with a bedding of sawdust and hay, various shelters, cardboard boxes and tree branches as enrichment. They received custom-made small desert rodent chow with insect and mineral supplements and fruits and vegetables *ad libitum* as a source of water. All study animals were descendants of 8 animals (5 males and 3 females), obtained by Moscow Zoo in December 2007 from a natural population in Egypt.

### Experimental procedure and USV recording

All acoustic recordings were conducted in a separate room where no other animals were present, at room temperature 23–28°C (mean ± SD = 25.1 ± 2.4) during daytime, at the same level of background noise. For USV recordings (sampling rate 384 kHz, 16 bit resolution) we used a Pettersson D1000X recorder with built-in microphone (Pettersson Electronik AB, Uppsala, Sweden). The microphone was established stationary at distance 15 cm above the tested animal. The obtained recordings had a high signal/noise ratio, the reverberation practically lacked.

Both pups and adults were tested singly. In total, each individual pup participated in 3 experimental trials (one trial per pup per age), at ages of 4–5, 6–7, and 8–9 days after birth; for details see [39]. Each individual adult participated in one trial per animal. Immediately before an experimental trial, the focal pup was taken from the nest and transferred in a small clean plastic hutch to the experimental room within the same floor of the building. Time from removal of the focal pup from the nest to the start of an experimental trial did not exceed 60 s. The experimental trial started, when the focal animal was placed to the experimental setup. Duration of each experimental trial was 420 s (7 min). Each trial took place in three stages: the isolation stage (120 s) followed by the touch stage (105 s), handling stage (105 s) and measurement stage (120 s). The duration of tests in this study was within range typical for those used in medical tests with rodent pups, 2–15 min [104].

For the duration of the isolation stage, a focal animal was located either in a clean plastic hutch (190x130x70 mm for pups) or in a plastic cylinder without bottom (diameter 193 mm, high 170 mm for adults), standing on even plastic table surface. Both the plastic huge and cylinder were open from above, i.e. from the side where the microphone was placed. For the duration of the touch stage, the experimenter (ASZ) gently touched the focal animal with a cotton bug, approximately two times per secondу. For the duration of the handling stage, the experimenter took the focal animal in hands and rotated it following [105] on its back. For the duration of the measurement stage, the experimenter thrice measured body length, head length, foot length the tail length with an electronic caliper, continuing keeping it in hands; the measurements were used in the study [84]. The end of measurements was the end of the trial. Although the experimenter hand surface temperature (28–30°C, [106]) was slightly higher than the temperature in the experimental room, a pup was held by fingers, so a possibility of additional warming the pup from the hand lacked. In contrast to pups, the adults were handheld during the handling and measurement stages.

After the end of a trial, the focal pup was placed to a heating hutch with a bedding of a cotton fabric, standing in the neighboring room. Experimental trials with all littermates were done consequently in the same manner. Then all the litter in total was returned to their home cage to their parents; the time of pup stay out of the nest did not exceed 40 min. Although pups were not individually identified, the sequential trials with littermates allowed controlling that each pup participated in experiments only once per age. The adults were taken from their home cages before experiments with a clean plastic glass and returned to the cage after the test trial. The experimental setup was rubbed with napkin wetted with alcohol after each experimental trial, to avoid effect of smell on USV of the next focal animal in the next experimental trial [68,107]. Each trial was recorded as a wav-file.

### Call samples

Visual inspection of spectrograms of acoustic files using Avisoft SASLab Pro software (Avisoft Bioacoustics, Berlin, Germany) showed that USV calls lacked in the study sample of fat-tailed gerbil pups until the 5^th^ day of life [39]. Therefore, we selected for analysis only the acoustic files recorded from 5^th^ to 9^th^ day of pup life. For some pups at some ages, recording trials were missing by refusals of equipment or for other reasons. As a result, 110 trials of the potential 120 (40 pups at 3 ages) recording trials were included in analysis. For the 20 study adults, we included in the visual inspection all available 20 files corresponding to the trials, one file per adult individual.

Visual inspection of spectrograms of pup acoustic files revealed that only 37 of the 110 audio files contained USV calls (4 of the 38 files for the 5-d pups, 11 of the 37 files for the 7-d pups and 22 of the 35 files for the 9-d pups). In total, USV calls were available for 24 of the 40 study pups, from 10 of the 11 litters; 13 of the 37 audio files were from repeated recording trials of the same pups at different ages. For the 11^th^ litter, pup USV calls were only available since 10^th^ day of pup life, therefore we did not include it in the analysis.

Visual inspection of spectrograms of adult acoustic files revealed that only 7 of the 20 audio files contained USV calls. Therefore, the USV calls were available from 7 individual adults (3 males and 4 females).

For analyses, we took all measurable (high quality) USV syllables of pups and adults. The USV syllables contained from one to a few notes. We defined a note as USV contour either continuous without breaks or with breaks shorter 10 ms and frequency jumps less than 10 kHz. We defined a syllable as one to a few USV notes separated with frequency jumps over 10 kHz [7,8]. If the separation break between notes exceeded 10 ms, we considered that the notes belong to different syllables. This syllable separation criterion was adjusted after [10], applied the 12.75-ms syllable separation criterion for USV syllables in domestic mice.

For pups, we measured in total 782 USV syllables (from 3 to 102 USV syllables per trial, on average 21.1±22.5 USV syllables per trial). For adults, we measured in total 232 USV syllables (from 14 to 90 syllables per trial, on average 33.1±26.0 USV per trial). For 5-d pups, we analysed 17 USV syllables from 4 trials; for 7-d pups 177 USV syllables from 11 trials; for 9-d pups 588 USV syllables from 22 trials.

### Acoustic analysis

Measurements of acoustic variables of pup and adult USV syllables have been conducted with Avisoft SASLab Pro software and exported to Microsoft Excel (Microsoft Corp., Redmond, WA, USA). As minimum fundamental frequency (f0min) of USV calls always exceeded 10 kHz, before measurements all wav-files were subjected to 10 kHz high-pass filtering, to remove low-frequency noise.

**Fig 1:**
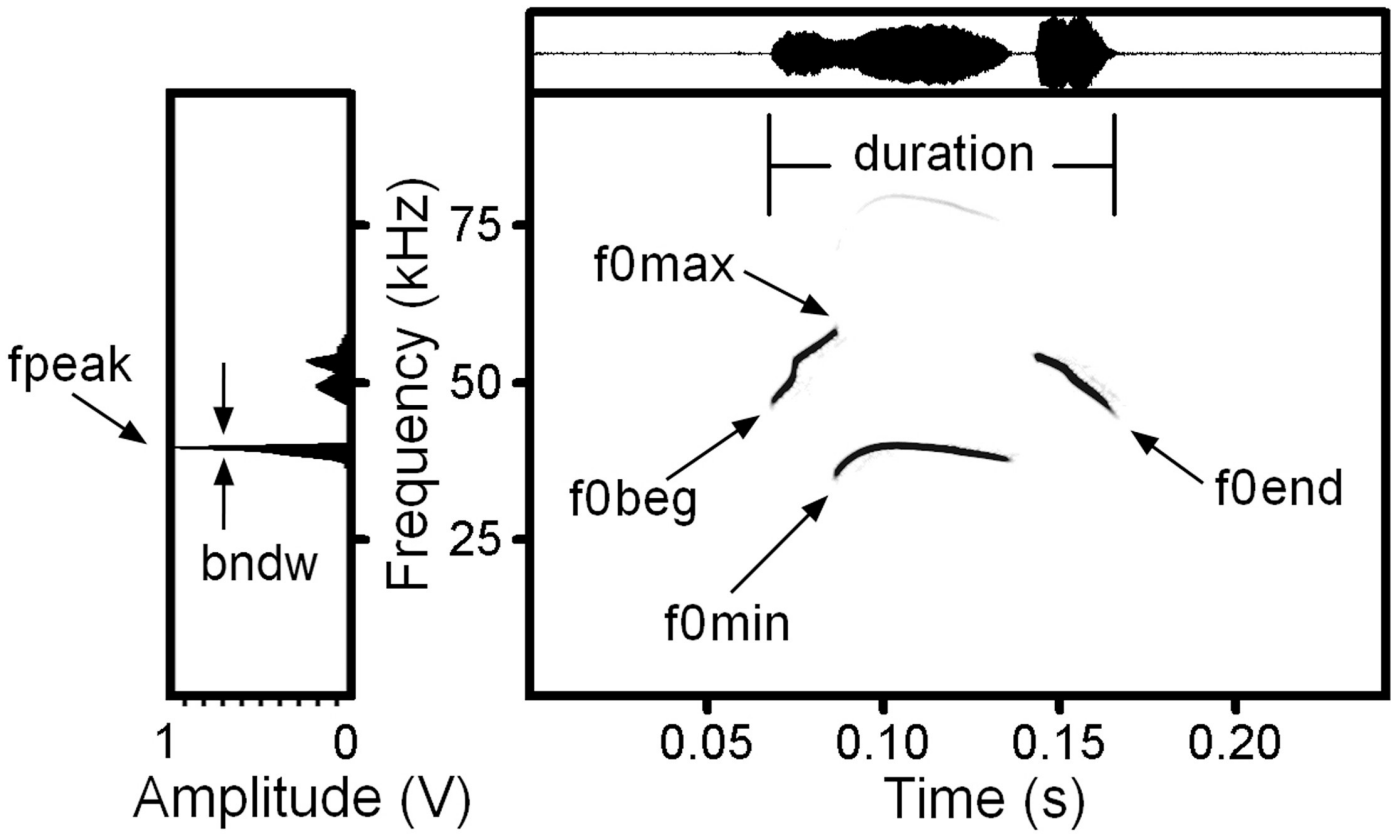
Measured variables for fat-tailed gerbils USV syllables exemplified by a pup 3-note down-up USV syllable with Chevron contour. Spectrogram (right) and mean power spectrum of the entire call (left). Designations: duration–syllable duration; f0beg–the fundamental frequency at the onset of a call; f0end–the fundamental frequency at the end of a call; f0max–the maximum fundamental frequency; f0min–the minimum fundamental frequency; fpeak–the frequency of maximum amplitude within a syllable; bndw–the bandwidth of the fpeak at the distance of 10 dB from the maximum Spectrogram was created using sampling frequency 192 kHz Hamming window, FFT 1024 points, frame 50% and overlap 93.75%.

For each USV syllable, we measured, in the spectrogram window of Avisoft (sampling frequency 384 kHz, Hamming window, FFT 1024 points, frame 50%, overlap 93.75%, providing frequency resolution 375 Hz and time resolution 0.17 ms), the duration with the standard marker cursor, and the maximum fundamental frequency (f0max), the minimum fundamental frequency (f0min), the start fundamental frequency (f0beg) and the end fundamental frequency (f0end) with the reticule cursor (Fig 1 and S1 Table). For each USV syllable, we measured, in the power spectrum window of Avisoft, the frequency of maximum amplitude (fpeak) from the syllable’s mean power spectrum and the bandwidth (bndw) of the fpeak at the distance of 10 dB from the maximum (Fig 1 and S1 Table).

### USV syllable types

**Fig 2:**
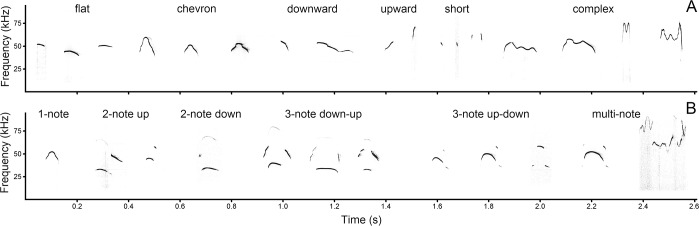
**Six contour shapes (A, upper panel) and six note compositions (B, lower panel).** Based on their combinations, USV syllables of fat-tailed gerbils were classified to distinct types (S1 Audio). Spectrogram was created using sampling frequency 192 kHz Hamming window, FFT 1024 points, frame 50%, overlap 87.5%.

In the spectrogram window of Avisoft, we classified USV syllables accordingly to the six possible f0 contour shapes: flat, chevron, downward, upward, short, complex (Fig 2 and S1 Audio). This classification was based (with modifications) on classifications developed for domestic mice by [6–8]. The flat contour was denoted when the difference between f0min and f0max was less than 6 kHz [7]. The short contour was denoted when the duration was shorter 4 ms [6,7]. In addition, when the difference between f0min and f0max exceeded 6 kHz, the denoted syllable contours could be the chevron (up-down one time), downward (descending from start to end), upward (ascending from start to end) or complex (up-down many times).

In addition to classifying by contour shape, we classified the USV syllables accordingly to the six possible note compositions (1-note, 2-note up, 2-note down, 3-note down-up, 3-note up-down, multi-note) based on the number of notes within syllable and presence of up or/and down frequency jumps over 10 kHz (Figs 1 and 2). The 1-note syllables lacked frequency jumps; the 2-note up syllables had one frequency jump up; the 2-note down syllables had one frequency jump down; the 3-note down-up syllables had two frequency jumps, first down and then up; the 3-note up-down syllables had two frequency jumps, first up and then down; and the multi-note syllables had three or more frequency jumps (Fig 2 and S1 Audio).

### Statistical analyses

Statistical analyses were made with STATISTICA, v. 8.0 (StatSoft, Tulsa, OK, USA), all means are given as mean ± SD. Significance levels were set at 0.05, and two-tailed probability values are reported. We used a nested design ANOVA with individual nested in age (with age as fixed factor and individual as random factor) to compare USV variables between pups and adults. We used one-way ANOVA with Tukey HSD (Honestly Significant Difference) test to estimate the effect of syllable contour shape and syllable composition on USV variables of pups and adults. In the case when not all sample sizes fitted to ANOVA assumptions for inclusion in analysis of groups differing in size not more than ten times, we calculated ANOVA results both for all groups in total and separately for the groups fitting this ANOVA assumptions.

## Results

### Types of USV syllables in pups and adults

Of the 36 distinct USV syllable types potentially possible by combining the six syllable contours and six note compositions, in pups we detected only 18 types and in adults only 24 types; 16 types were shared by pups and adults (Tables 1 and 2). In pups, most frequent was the 1-note syllable (600 of the total 782 USV syllables) with contours either flat (299 USV syllables) or chevron (193 USV syllables). Another frequent type in pups was the 3-note down-up syllable type with the contour chevron (96 USV syllables) (Table 1). In adults, as in pups, most frequent was the 1-note syllable (178 of the total 232 USV syllables) with the contours chevron (55 USV syllables), flat (34 USV syllables) or short (32 USV syllables) (Table 2). In addition, two USV syllables of two individual pups and four USV syllables of two individual adults contained the nonlinear phenomenon biphonation (interaction between the USV fundamental frequency and the audible fundamental frequency).

**Table 1:** Number and percentage of 18 distinct USV syllable types of pups, classified based on combination of contour shape and note composition.

Note composition	Number pups, litters	Contour shape						Total USV syllables
		flat	chevron	downward	upward	short	complex	
		23 pups, 9 litters	22 pups, 9 litters	18 pups, 9 litters	8 pups, 8 litters	6 pups, 4 litters	4 pups, 3 litters	
1-note	24 pups, 10 litters	299	193	87	6	9	6	600 (76.73%)
2-note up	7 pups, 7 litters	7	16	7	0	0	0	30 (3.84%)
2-note down	8 pups, 6 litters	11	16	0	3	0	0	30 (3.84%)
3-note down-up	12 pups, 7 litters	0	96	0	0	0	0	96 (12.28%)
3-note up-down	3 pups, 3 litters	1	17	4	0	0	0	22 (2.81%)
multi-note	3 pups, 3 litters	0	2	0	1	0	0	4 (0.51%)
Total USV syllables		318 (40.66%)	340 (43.48%)	99 (12.66%)	10 (1.28%)	9 (1.15%)	6 (0.77%)	782 (100%)

**Table 2:** Number and percentage of 24 distinct USV syllable types of adults, classified based on combination of contour shape and note composition.

Note composition	Number adults	Contour shape						Total USV syllables
		flat	chevron	downward	upward	short	complex	
		7 adults	7 adults	7 adults	7 adults	5 adults	6 adults	
1-note	7 adults	34	55	25	26	32	6	178 (76.72%)
2-note up	6 adults	2	9	2	2	0	1	16 (6.90%)
2-note down	6 adults	6	2	3	3	0	4	18 (7.79%)
3-note down-up	4 adults	0	3	1	0	0	2	6 (2.59%)
3-note up-down	4 adults	1	3	0	0	0	0	4 (1.72%)
multi-note	5 adults	0	6	1	0	0	3	10 (4.31%)
Total USV syllables		43 (18.53%)	78 (33.62%)	32 (13.79%)	31 (13.36%)	32 (13.79%)	16 (6.90%)	232 (100%)

**Fig 3:**
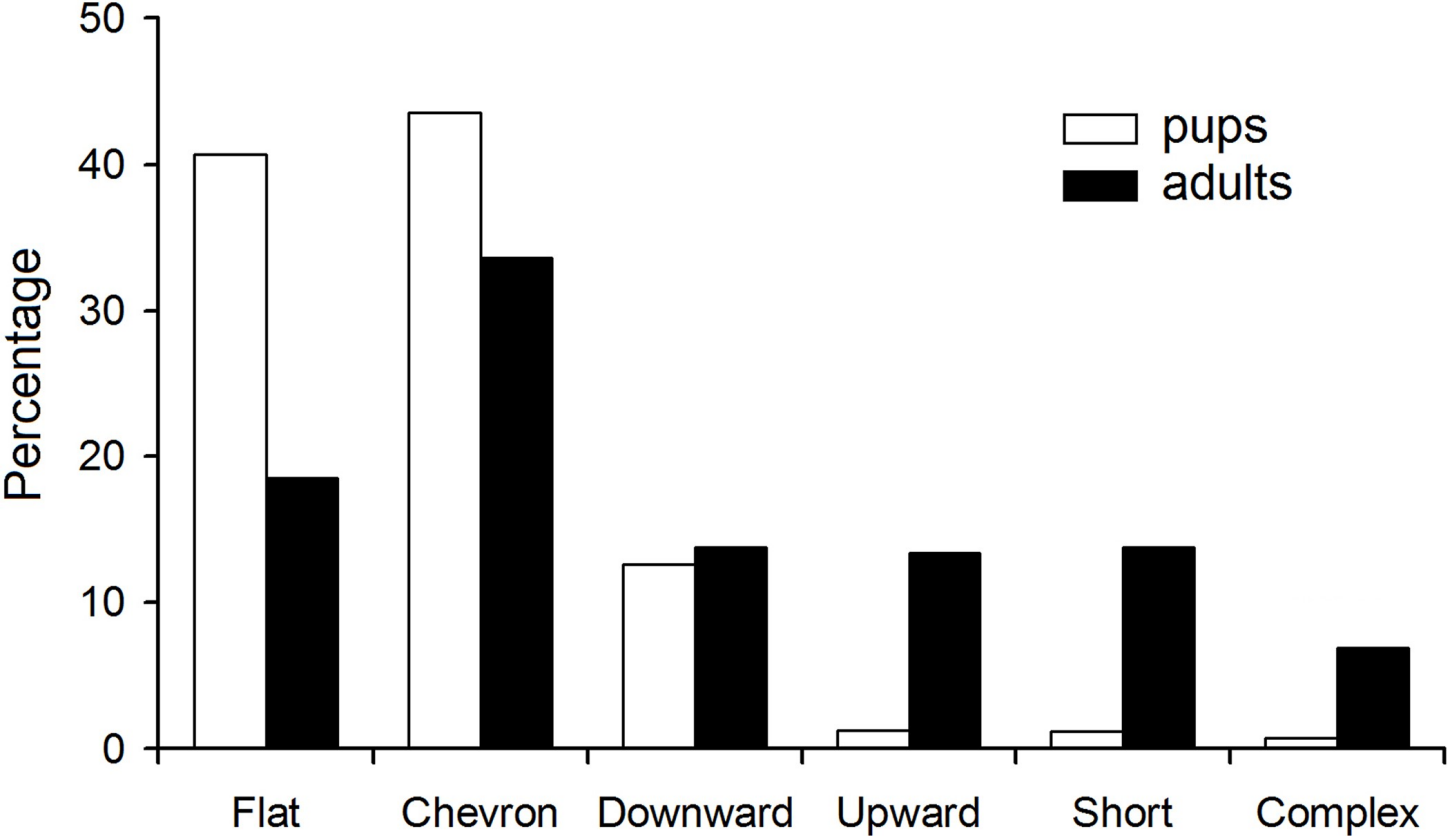
Percentages of pup and adult USV syllables with different contour shapes.

The contours flat and chevron were more frequent in pups than in adults (Fig 3). The contours upward, short and complex were more frequent in adults than in pups. The downward contour equally frequently occurred in pups and adults (Fig 3).

**Fig 4:**
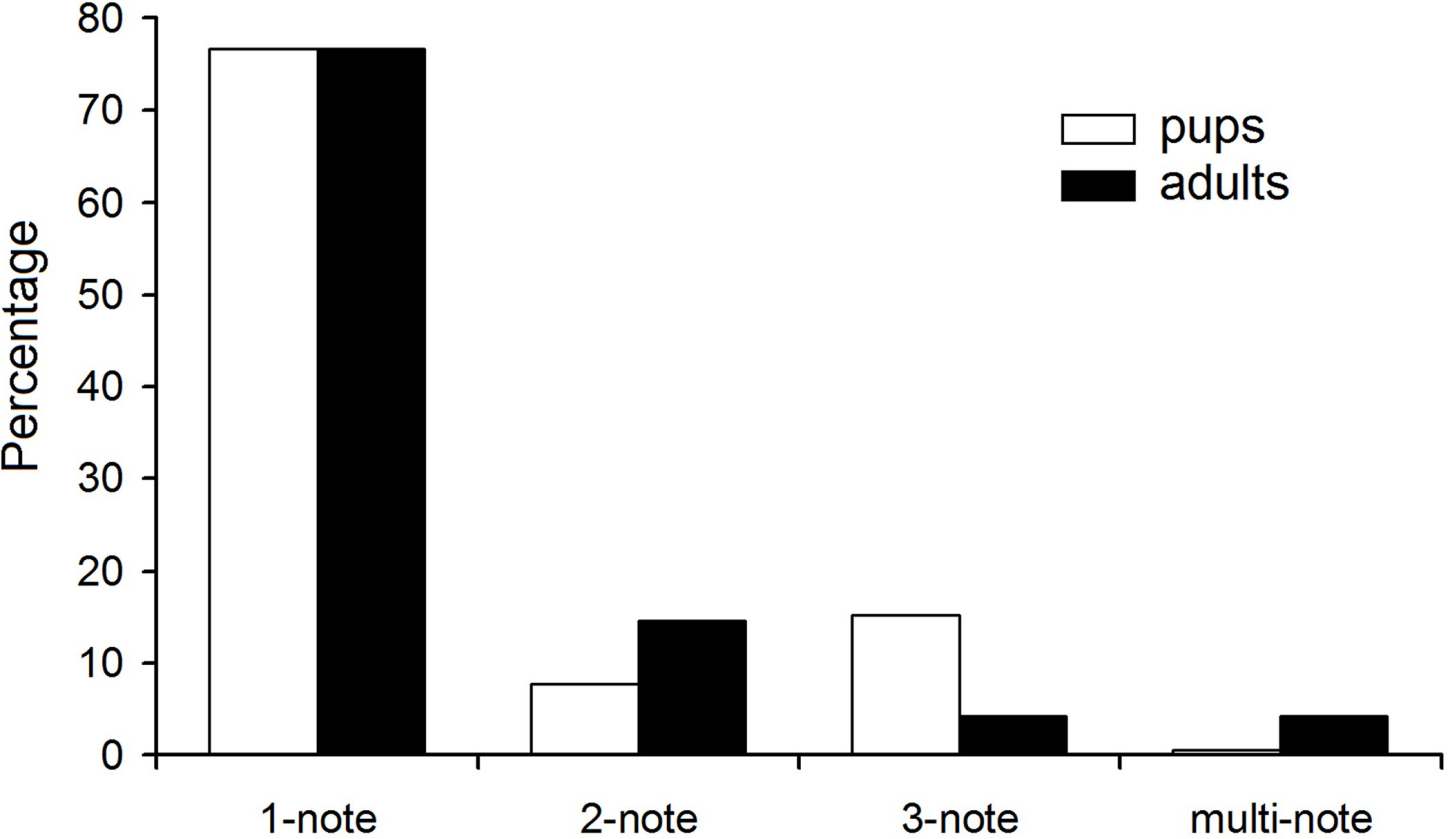
Percentages of pup and adult USV syllables with different note compositions.

Percentages of the most frequent 1-note syllables were the same in pups and adults (Fig 4). The 2-note syllables were more frequent in adults than in pups, whereas the 3-note syllables were more frequent in pups than in adults and the multi-note syllables were more frequent in adults than in pups.

### Acoustic variables of pup and adult USV syllables

On the total sample of USV syllables of all types, the syllable duration was found longer in pups than in adults, whereas the fpeak, bandw and all f0 variables were lower in pups than in adults (Table 3). Similar results for comparisons between pups and adults were obtained on separately taken samples of all 1-note USV syllables (Table 4) and separately for flat 1-note USV syllables (Table 5) and chevron 1-note USV syllables (Table 6).

**Table 3:** Values (mean±SD, min-max) of acoustic variables of pup and adult USV syllables and nested ANOVA results for their comparison.

Acoustic variable	Pups (*n* = 782 USV syllables)	Adults (*n* = 232 USV syllables)	ANOVA
duration (ms)	50.0±31.0 (2.4–154.7)	22.0±32.7 (1.7–354.3)	*F*_1,983_ = 81.2, *p*<0.001
f0max (kHz)	52.2±5.7 (32.6–120.0)	66.8±13.9 (23.6–113.3)	*F*_1,983_ = 367.8, *p*<0.001
f0min (kHz)	41.9±6.7 (19.1–80.6)	51.1±9.6 (18.4–97.9)	*F*_1,983_ = 136.2, *p*<0.001
f0beg (kHz)	47.1±5.7 (19.5–120.0)	57.3±10.2 (21.8–103.2)	*F*_1,983_ = 186.8, *p*<0.001
f0end (kHz)	44.6±5.5 (29.6–80.2)	55.7±13.4 (18.4–113.3)	*F*_1,983_ = 319.9, *p*<0.001
fpeak (kHz)	47.9±6.1 (30.7–87.7)	60.0±10.5 (20.6–108.3)	*F*_1,983_ = 262.9, *p*<0.001
bndw (kHz)	3.1±1.5 (1.5–20.6)	6.5±6.1 (1.8–31.8)	*F*_1,983_ = 114.5, *p*<0.001

**Table 4:** Values (mean±SD) of acoustic variables of fat-tailed gerbil pup and adult 1-note USV syllables and nested ANOVA results for their comparison.

Acoustic variable	Pups (*n* = 600 USV syllables)	Adults (*n* = 178 USV syllables)	ANOVA
duration (ms)	41.8±27.2	16.6±29.3	*F*_1,747_ = 59.9, *p*<0.001
f0max (kHz)	50.9±5.2	63.8±11.5	*F*_1,747_ = 230.0, *p*<0.001
f0min (kHz)	44.7±4.5	52.3±9.6	*F*_1,747_ = 214.1, *p*<0.001
f0beg (kHz)	47.6±5.5	57.5±9.8	*F*_1,747_ = 130.5, *p*<0.001
f0end (kHz)	45.3±4.8	55.1±11.9	*F*_1,747_ = 109.8, *p*<0.001
fpeak (kHz)	49.2±4.4	60.1±9.3	*F*_1,747_ = 241.6, *p*<0.001
bndw (kHz)	3.0±1.3	6.1±5.6	*F*_1,747_ = 79.3, *p*<0.001

**Table 5:** Values (mean±SD) of acoustic variables of fat-tailed gerbil pup and adult flat 1-note USV syllables and nested ANOVA results for their comparison.

Acoustic variable	Pups (*n* = 299 USV syllables)	Adults (*n* = 34 USV syllables)	ANOVA
duration (ms)	30.2±19.5	19.3±13.0	*F*_1,304_ = 4.99, *p* = 0.03
f0max (kHz)	49.0±4.4	59.1±8.1	*F*_1,304_ = 117.8, *p*<0.001
f0min (kHz)	45.8±4.3	54.8±7.6	*F*_1,304_ = 112.2, *p*<0.001
f0beg (kHz)	47.5±4.5	56.4±7.3	*F*_1,304_ = 95.1, *p*<0.001
f0end (kHz)	46.3±4.5	56.0±8.6	*F*_1,304_ = 126.4, *p*<0.001
fpeak (kHz)	48.0±4.3	58.2±7.7	*F*_1,304_ = 125.4, *p*<0.001
bndw (kHz)	2.4±0.6	2.7±0.8	*F*_1,304_ = 7.52, *p* = 0.007

**Table 6:** Values (mean±SD) of acoustic variables of fat-tailed gerbil pup and adult chevron 1-note USV syllables and nested ANOVA results for their comparison.

Acoustic variable	Pups (*n* = 193 USV syllables)	Adults (*n* = 55 USV syllables)	ANOVA
duration (ms)	64.8±23.5	17.8±14.3	*F*_1,222_ = 89.5, *p*<0.001
f0max (kHz)	52.5±3.3	65.8±11.2	*F*_1,222_ = 26.6, *p*<0.001
f0min (kHz)	43.1±3.2	50.2±8.7	*F*_1,222_ = 4.09, *p* = 0.04
f0beg (kHz)	45.4±3.3	56.8±8.9	*F*_1,222_ = 34.7, *p*<0.001
f0end (kHz)	43.8±3.3	50.6±8.9	*F*_1,222_ = 4.32, *p* = 0.04
fpeak (kHz)	50.3±3.3	61.4±9.1	*F*_1,222_ = 22.7, *p*<0.001
bndw (kHz)	3.5±1.3	7.6±6.5	*F*_1,222_ = 9.25, *p* = 0.003

Unexpectedly, although pup calls were overall longer by duration and lower-frequency than in adults, the longest USV syllable was found in an adult individual. Similarly, the USV syllable with the maximal value of f0 was found in a pup individual (Table 3).

In pup 1-note USV syllables, the syllable contour shape affected all acoustic variables (Table 7). Compared to other syllable contour shapes, the USV syllables with the complex contour had the longest duration and the lowest f0min and f0beg. The USV syllables with the short contour had the shortest duration and the lowest f0max, fpeak and bndw. The USV syllables with the upward contour had the highest f0max, f0min, f0end and fpeak. The USV syllables with the downward contour had the highest f0beg and bndw. The USV syllables with the chevron contour had the lowest f0end, whereas the USV syllables with the flat contour had intermediate values of acoustic variables compared to other syllable contour shapes (Table 7). For USV syllables with three most frequently occurring contour shapes (flat, chevron and downward), one-way ANOVA results with Tukey HSD test coincided with results of their comparison in Table 7.

**Table 7:** Values (mean±SD) of acoustic variables of pup 1-note USV syllables with different contour shapes (flat, chevron, downward, upward, short, complex) and one-way ANOVA results for their comparison.

Acoustic variable	Contour shape						ANOVA
	flat	chevron	downward	upward	short	complex	
duration (ms)	30.2±19.5 ^a^	64.8±23.5 ^b^	32.5±18.6 ^a^	19.7±14.9 ^a,c^	3.7±0.5 ^c^	97.2±38.1 ^d^	*F*_5,594_ = 84.4, *p*<0.001
f0max (kHz)	49.0±4.4 ^a,d^	52.5±3.3 ^b^	53.5±7.7 ^b,c^	58.2±8.1 ^c^	46.6±7.5 ^a^	54.1±3.2 ^b,c,d^	*F*_5,594_ = 23.0, *p*<0.001
f0min (kHz)	45.8±4.3 ^a,c^	43.1±3.2 ^b^	44.2±5.7 ^b^	50.7±7.7 ^c^	44.7±7.0 ^a,b,c^	41.2±4.9 ^a,b^	*F*_5,594_ = 12.2, *p*<0.001
f0beg (kHz)	47.5±4.5 ^a^	45.4±3.3 ^b^	53.0±7.7 ^c^	50.8±8.0 ^a,b,c^	46.3±7.4 ^a,b^	43.4±6.6 ^a,b^	*F*_5,594_ = 31.0, *p*<0.001
f0end (kHz)	46.3±4.5 ^a^	43.8±3.3 ^b^	44.3±5.7 ^b^	57.8±8.6 ^c^	45.0±7.1 ^a,b^	44.0±4.0 ^a,b^	*F*_5,594_ = 17.6, *p*<0.001
fpeak (kHz)	48.0±4.3 ^a^	50.3±3.3 ^b^	50.5±4.8 ^b^	55.1±7.3 ^b^	45.8±7.2 ^a^	50.7±3.0 ^a,b^	*F*_5,594_ = 12.9, *p*<0.001
bndw (kHz)	2.4±0.6 ^a^	3.5±1.3 ^b^	4.3±1.8 ^c^	2.7±0.6 ^a,b^	2.3±0.6 ^a^	3.6±1.8 ^a,b,c^	*F*_5,594_ = 50.8, *p*<0.001

Note: The same superscripts indicate which values did not differ significantly (*p*>0.05, Tukey HSD test).

In adults, distinctive to pups, the contour shape affected not all acoustic variables of 1-note USV syllables, but only the duration, f0beg, f0end and bndw (Table 8). Compared to other syllable contour shapes, the USV syllables with the complex contour had the longest duration and the highest f0max and fpeak. The USV syllables with the short contour had the shortest duration. The USV syllables with the upward contour had the highest f0end and the lowest f0beg. The USV syllables with the downward contour had the highest f0beg and bndw (as in pups) and the lowest f0min. The USV syllables with the chevron contour had the lowest f0end. The USV syllables with the flat contour had the highest f0min and the lowest f0max, fpeak and bndw (Table 8).

**Table 8:** Values (mean±SD) of acoustic variables of adult 1-note USV syllables with different contour shapes (flat, chevron, downward, upward, short, complex) and one-way ANOVA results for their comparison.

Acoustic variable	Contour shape						ANOVA
	flat	chevron	downward	upward	short	complex	
duration (ms)	19.3±13.0 ^a^	17.8±14.3 ^a^	10.7±7.2 ^a^	14.3±15.6 ^a^	3.2±0.6 ^a^	95.1±129.2 ^b^	*F*_5,172_ = 14.1, *p*<0.001
f0max (kHz)	59.1±8.1	65.8±11.2	65.4±15.2	64.8±9.6	62.6±13.1	67.8±7.4	*F*_5,172_ = 1.9, *p* = 0.10
f0min (kHz)	54.8±7.6	50.2±8.7	49.5±12.2	54.4±7.4	53.8±11.9	51.2±5.7	*F*_5,172_ = 1.9, *p* = 0.10
f0beg (kHz)	56.4±7.3 ^a^	56.8±8.9 ^a,b^	63.0±13.6 ^a^	54.5±7.4 ^b^	57.8±11.2 ^a,b^	57.2±2.4 ^a,b^	*F*_5,172_ = 2.4, *p* = 0.04
f0end (kHz)	56.0±8.6 ^a,b,c^	50.6±8.9 ^a^	50.7±14.6 ^a,c^	64.1±9.6 ^b,c^	57.7±14.9 ^b,c^	56.2±6.2 ^a,b,c^	*F*_5,172_ = 6.4, *p*<0.001
fpeak (kHz)	58.2±7.7	61.4±9.1	60.0±11.1	60.2±7.0	59.3±11.9	62.2±6.7	*F*_5,172_ = 0.6, *p* = 0.70
bndw (kHz)	2.7±0.8 ^a^	7.6±6.5 ^b,c^	9.3±7.9 ^c^	4.7±3.0 ^a,b^	5.7±4.4 ^a,b,c^	7.1±3.7 ^a,b,c^	*F*_5,172_ = 6.1, *p*<0.001

Note: The same superscripts indicate which values did not differ significantly (*p*>0.05, Tukey HSD test).

Number of notes within USV syllable affected all USV acoustic variables for the exclusion of bndw in pups (Table 9) and for the exclusion of f0beg and fpeak in adults (Table 10). In both pups and adults, the highest values of duration and f0max were found in the 3-note and in the multi-note syllables (Tables 9 and 10). In both pups and adults, the shortest duration, the lowest f0max and the highest f0min were found in the 1-note USV syllables (Tables 9 and 10). The intermediate values of acoustic variables were found in the 2-note USV syllables.

**Table 9:** Values (mean±SD) of acoustic variables of pup USV syllables with different number of notes within syllable (1, 2, 3, multi-note) and one-way ANOVA results for their comparison.

Acoustic variable	Number of notes within USV syllable				ANOVA
	1-note	2-note	3-note	multi-note	
duration (ms)	41.8±27.2 ^a^	58.6±26.3 ^b^	86.5±22.9 ^c^	68.0±33.7 ^a,b,c^	*F*_3,778_ = 96.1, *p*<0.001
f0max (kHz)	50.9±5.2 ^a^	56.3±7.0 ^b^	56.7±3.1 ^b^	59.4±1.9 ^b^	*F*_3,778_ = 59.5, *p*<0.001
f0min (kHz)	44.7±4.5 ^a^	34.9±5.4 ^b^	31.6±2.2 ^c^	33.9±1.9 ^b,c^	*F*_3,778_ = 367.6, *p*<0.001
f0beg (kHz)	47.6±5.5 ^a^	44.4±7.9 ^b^	46.1±4.9 ^b^	39.9±8.4 ^b^	*F*_3,778_ = 9.5, *p*<0.001
f0end (kHz)	45.3±4.8 ^a^	42.9±9.3 ^b^	42.0±5.3 ^b^	41.0±10.3 ^a,b^	*F*_3,778_ = 15.4, *p*<0.001
fpeak (kHz)	49.2±4.4 ^a^	44.7±9.4 ^b^	43.2±8.1 ^b^	43.0±8.5 ^a,b^	*F*_3,778_ = 46.1, *p*<0.001
bndw (kHz)	3.0±1.3	3.1±2.6	3.3±1.5	2.9±0.3	*F*_3,778_ = 1.1, *p* = 0.34

Note: The same superscripts indicate which values did not differ significantly (*p*>0.05, Tukey HSD test).

**Table 10:** Values (mean±SD) of acoustic variables of adult USV with different number of notes within syllable (1, 2, 3, multi-note) and one-way ANOVA results for their comparison.

Acoustic variable	Number of notes within USV syllables				ANOVA
	1-note	2-note	3-note	multi-note	
duration (ms)	16.6±29.3 ^a^	31.3±31.0 ^b^	45.7±25.0 ^b,c^	64.1±54.6 ^c^	*F*_3,228_ = 11.1, *p*<0.001
f0max (kHz)	63.8±11.5 ^a^	75.3±18.1 ^b^	81.5±14.2 ^b^	76.6±11.3 ^b^	*F*_3,228_ = 14.5, *p*<0.001
f0min (kHz)	52.3±9.6 ^a^	47.8±8.4 ^b^	48.3±11.8 ^a,b^	43.2±4.7 ^b^	*F*_3,228_ = 5.0, *p* = 0.002
f0beg (kHz)	57.5±9.8	55.5±13.0	59.8±8.5	57.7±7.9	*F*_3,228_ = 0.6, *p* = 0.62
f0end (kHz)	55.1±11.9 ^a,b^	61.2±18.3 ^a^	54.4±15.5 ^a,b^	49.1±12.9 ^b^	*F*_3,228_ = 2.9, *p* = 0.03
fpeak (kHz)	60.1±9.3	59.4±14.7	61.2±11.8	58.1±12.9	*F*_3,228_ = 0.2, *p* = 0.91
bndw (kHz)	6.1±5.6 ^a^	6.0±5.6 ^a^	11.5±10.6 ^b^	10.2±8.1 ^a,b^	*F*_3,228_ = 4.0, *p* = 0.009

Note: The same superscripts indicate which values did not differ significantly (*p*>0.05, Tukey HSD test).

## Discussion

### General findings

This study revealed a rich repertoire of ultrasonic syllables in fat-tailed gerbils. Based on contour shape and note composition, we identified 24 distinct USV syllable types in adults and 18 in pups; 16 of these 26 types were shared by adults and pups. Percentages of syllables with certain number of notes were similar between pups and adults. In both pups and adults, 1-note syllables were most common. The prevalence of 1-note USV calls was also reported in hamsters [108,109].

In fat-tailed gerbils, pup USV syllables differed from adult USV syllables by the occurrence of different contour shapes. Similarly, the developmental changes in the proportions of different syllable types were reported for domestic mice [7], Norway rats [35,110] and *Scotinomys* singing mice [67]. Age-related differences in the proportion of 22-kHz and 50-kHz calls were reported in rats [111].

Overall, pup USV syllables were of longer duration and lower-frequency than adult USV syllables. The USV syllable contour shape and note composition significantly affected most temporal, frequency and power variables in both pups and adults, similarly with findings in domestic mice [7,112].

In fat-tailed gerbils, the overall USV fundamental frequency range was from 18 kHz to 120 kHz, and the duration of USV calls ranges from 2 ms to 350 ms. This frequency range is comparable with those reported in rats, from 20 kHz to over 90 kHz, whereas the duration range of USV calls in rats is different, from approximately 10 ms to over 3500 ms [113].

### No evidence for vocal learning in fat-tailed gerbils

In mammals whose vocal repertoires are assumed to be fixed at birth, the same call types can be found in pups and adults [114,115]. Our results confirm therefore that ultrasonic vocalizations of fat-tailed gerbils are innate, as the same 16 USV syllables occurred in both pups and adults. Similar findings were obtained for other rodents and insectivores. In *Scotinomys* singing mice, their ultrasonic long FM down-sweeps that comprise adult advertisement song were produced from birth [67]. In the piebald shrew *Diplomesodon pulchellum*, seven of the eight call types were shared by pups and adults [116,117]. In the Asian house shrew *Suncus murinus*, five of seven call types found in pups were also found in adults [118]. In rats, creation of strains with high or low rates of isolation-induced USV evokes corresponding changes in ontogeny of acoustic parameters, suggesting that traits of USV calls are genetically predetermined [119]. In domestic mice, pups produced 10 of 11 USV syllable types recorded from adults [7].

In addition, in domestic mice, embryo-transfer and cross-fostering experiments suggest that USV calls are innate [47,120,121]. Research suggests that Foxp2 gene plays a crucial role in vocal development in mammals [122] and in particular in mice [123]. In mice, the chevron and short contours of USV syllables are coupled respectively with two genotypically different strains and are inherited by the Mendelian law of independent assortment [124].

We should point however, that in spite of this apparently very strict genetic control of vocalization, another study reports some level of developmental plasticity of mice vocalization [8]. This level of plasticity is well comparable to those in mammals with innate vocal repertoires vocalizing in the audible range of frequencies [125–127].

### USV call types in rodent species

The overall richness of USV syllable types, found in fat-tailed gerbils in this study (26 distinct calls types), was comparable with those reported for bats (28 distinct call types) [128,129]. The acoustic variation of USV syllable types in pup fat-tailed gerbils (18 USV syllable types) was comparable with level of variation (10–12 USV syllable types) in pup domestic mice [6–8,50,130] and in pup Norvay rats [16,35,131]. At the same time, seven USV call types were identified in pup short-tailed field voles *Microtus agrestis* [132]. Six USV call types were identified in pup *Scotinomys* [67] and in pup Mongolian gerbils [133,134]. In *Peromyscus* pups, the three identified USV call types were shared with adults but did not have clear boundaries between the types and could grade into one another [102]. Two USV call types were identified in pup Djungarian humsters *Phodopus sungarus* [99]. One USV call type was identified in pup Syrian hamsters *Mesocricetus auratus*, in pup Chinese hamsters *Cricetulus griseus* [99], in pup Key Largo woodrats *Neotoma floridana smalli* [135], but further analyses most probable will identify more types.

Among adult rodents, 15 distinct USV call types were identified in Norway rats [77,136]; some of these call types were shared between the Norway and 8 other poorly investigated bioacoustically *Rattus* species [137]. Eight USV call types were identified in adult African woodland dormice *Graphiurus murinus* [138]. Five USV call types were identified in adult hazel dormice *Muscardinus avellanarius* [139]; 4 USV call types were identified in adult *Peromyscus* [140]. Three USV call types were identified in Mongolian gerbils [141], but these analyses were limited with sexual behaviour. One USV call type was identified in adult Key Largo woodrats [135].

Overall, the USV calls of fat-tailed gerbils displayed many similar acoustic traits with USV calls of domestic mice, Norway rats and other rodents. Therefore, from the applied perspective, mice USV databases [142] and the automated software for detection, clustering and analyses of mice USV syllables [10,143–145], can potentially be adapted, re-tuned or modified for calls of fat-tailed gerbils and other laboratory rodent participating in biomedical experiments. For example, frequency jumps are also characteristic for domestic mice [7,8,146–149], Norway rats [16,61], short-tailed field voles [132] and collared lemmings *Dicrostmyx groenlandicus* [95]. Short notes within 5 ms are also characteristic for domestic mice [6,7] and Norway rats [14]. The different USV syllable contour shapes of fat-tailed gerbils are also similar with those reported in mice [6–8,148], Norway rats [14,16] and in short-tailed field voles [132]. Therefore, for classifying USV calls of fat-tailed gerbils in this study, we mostly followed the categorization scheme of USV syllables developed for domestic mice [6–8,148]. Although the categorization scheme of bat USV syllables [128,129] was previously applied by for classifying USV syllables of Mongolian gerbils [78], we did not use it in this study, because the USV syllable contour shapes reported in bats are rather distinctive from those produced by fat-tailed gerbils.

### Ontogenetic changes in USV acoustic variables

In fat-tailed gerbils, pup USV syllables were in general longer in duration and lower in frequency than adult USV syllables, and each particular call type shared the general ontogenetic pathway. The general pathway of USV ontogeny in fat-tailed gerbils (increasing fundamental frequency and call shortening) is opposed to those reported for rats (decreasing fundamental frequency and call lengthening) and strongly reminiscent of bats. In rat, the initial broad range of 30–65 kHz of pup so called 40-kHz calls USV frequencies split in adults into two non-overlapping frequency ranges, of 22 kHz and 50 kHz calls [1,35,72,150]. In particular, rat pup USV syllables decrease in fundamental frequency during first 2–3 wk of life [16,41,151]. Then they suddenly increase in fundamental frequency on 14^th^ [152] or 30^th^ day of pup life [16,113], depending on rat strain, and split to the adult-like 22-kHz and 50-kHz calls [136]. Then, since approximately 4 wks up to senescence, the fundamental frequency decreases in both 22-kHz and 50-kHz calls, irrespectively to the USV syllable contour [153–155]. At the same time, duration of rat USV calls remains stable until 3 wks, then suddenly decreases [16] and increases again from 6 wks up to senescence [153–155].

In bats, the ontogenetic changes of USV variables are similar to fat-tailed gerbils in spite of a distinctive to rodents mechanism for production of bat USV calls, based on vibrations of the thin vocal membranes on their vocal folds [156,157]. Throughout maturation, bats produce USV calls of the same [158] or an increasingly high fundamental frequency [159–172], in spite of the growing larynx [173]. USV calls’ shortening from pups to adults has been reported in the pomona leaf-nosed bat *Hipposideros pomona* [166], in Asian particolored bat *Vespertilio sinensis* [167], in the big brown bat *Eptesicus fuscus* [170] and in the long-fingered bat *Myotis capaccinii* [172].

In mice, USV syllables also shorten as in fat tailed gerbils, but decrease in frequency and each call type can display a specific pattern of developmental changes [6,98,174–180]. In domestic mice, as in Norway rats, the developmental analyses are complicated, because the ontogenetic trends of USV acoustic variables are strain-specific, although generally follow the species-specific pattern [181]. In ontogeny of *Peromyscus* rodents, USV syllables shortened, whereas frequency changes inconsistently [182].

### USV and audible calls and hearing sensitivity

Fat-tailed gerbils have the extraordinarily inflated tympanic bullae [85–87]. It can be related with low-frequency hearing and potential detection of vibrations passing through their sandy substrate for location insects and approaching predators such as owls or snakes [183,184] Nevertheless, this study showed that pup and adult fat-tailed gerbils produce many various USV calls. Study of hearing sensitivity suggests that in fat-tailed gerbils, the hearing in the human audible range of frequencies is shifted to the lower-frequency range compared to other gerbils [85]. Data on ultrasonic hearing of fat-tailed gerbils are unavailable. At the same time, studies on domestic mice showed that neurons in the dorsal cochlear nucleus, designed for responsiveness to USV calls below 30 kHz, are also responsive to social USV calls over 50 kHz [185], probably because of sound distortion when passing cohlea [186]. Fat-tailed gerbils with their low-frequency sensitivity can serve a potential convenient model for testing this hypothesis of hearing distorted ultrasound by rodents.

There is a preliminary evidence that during the isolation and handling procedure, pup and adult fat-tailed gerbils produce also very low-frequency audible calls [39]. Vocalization in both audible and ultrasonic frequency ranges occurs during experimental isolation and handling procedures in many species of rodents [26,109]. Further research should investigate the acoustic structure and use of the audible calls produced by pup and adult fat-tailed gerbils during isolation and handling procedure and alternation in their use with the USV calls.

In gerbils, ontogenetic emergence of USV calls is ahead substantially the opening of ears and eyes. Pup Mongolian gerbils produce USV calls since the 1st day of pup life, with maximum of ultrasound emission in 2-6-day pups [33,96,174]. In pup fat-tailed gerbils, first USV calls emerge since 5th day of pup life, with maximum of ultrasound emission in 11-14-day pups [39]. At the same time, in gerbils, the ear opening occurs between 12 and 28 postnatal day depending on the species and the eye opening occurs between 14 and 24 postnatal day depending on the species, see review in [review in 84]. In pup fat-tailed gerbils, the ears open very late, at postnatal day 27, whereas the eyes open between 16 and 24 postnatal days [80,84]. As in rats and mice, pup isolation USV calls are directed to their mothers at situations when pup USV calls promote retrieval by the pups by their mothers [36].

This study provides an additional evidence about the wide acoustic variation of USV calls in pup and adult gerbils. These variable calls may potentially play an important role in different social contexts. Further studies are necessary to reveal the relationships between the acoustics structure and the attending behaviours in the fat-tailed gerbil.

## Supporting information

S1 Table Acoustic measurements of USV syllables of fat-tailed gerbils for describing the acoustics of call types and for estimating the effects of age, contour shape and note composition on the acoustic variables. (XLS)

S1 Audio USV syllables of fat-tailed gerbils. Order as on Fig 2. Sampling frequency of the acoustic file is 192 kHz.
(WAV)
